# Spontaneous Acute Subdural Hematoma Associated with Arachnoid Cyst and Intra-cystic Hemorrhage

**DOI:** 10.7759/cureus.3383

**Published:** 2018-09-28

**Authors:** Ryan Johnson, Abdul Amine, Hamad Farhat

**Affiliations:** 1 Neurosurgery, Advocate Bromenn Medical Center, Normal, USA; 2 Neurosurgery, Advocate Christ Medical Center, Oak Lawn, USA

**Keywords:** arachnoid cyst, spontaneous subdural hematoma, intra-cystic hemorrhage

## Abstract

Arachnoid cysts (ACs) are congenital, extra-axial lesions containing fluid similar to the composition of cerebrospinal fluid. Usually found incidentally, these lesions are observed with serial imaging to document their growth patterns and stability, and are then followed conservatively until clinical symptoms develop. Surgical options for symptomatic arachnoid cysts include cyst aspiration, cyst evacuation with fenestration into the subarachnoid space, and shunt procedures including cysto-peritoneal and cysto-ventricular shunts. Intra-cystic hemorrhage and subdural hematoma are rare and more emergent sequelae of ACs that may require an emergent craniotomy. This case report further documents a rare cause of spontaneous subdural hematoma, as well as serves as a pivot point for further discussion into whether continued neuroimaging surveillance in patients with ACs would prove to be beneficial.

## Introduction

Arachnoid cysts (ACs) are congenital, non-neoplastic, extra-axial, intra-arachnoid cystic lesions that contain fluid similar to the composition of cerebrospinal fluid [1-3]. Most of these lesions are found incidentally while undergoing neuroimaging for other unrelated problems; however, ACs are known to be a cause of headaches, increasing head circumference, and developmental delay in pediatric patients; rarely, patients may develop symptoms of weakness, seizures, and endocrinopathies, depending on the AC's location [1]. Fortunately, most ACs remain stable over the patient’s lifetime, while complete disappearance has also been reported [4]. The feared complication associated with ACs is the possibility of rapid expansion secondary to the intra-cystic hemorrhage or tearing of the subdural bridging veins with subsequent formation of a subdural hematoma (SDH), both of which can be life-threatening to the patient. The SDH can be spontaneous as the cyst continues to grow or, more commonly, can be the result of physical exertion or traumatic insult [1-3]. In this article, we present a case of an atraumatic spontaneous acute subdural hematoma associated with a previously deemed stable arachnoid cyst.  

## Case presentation

**Figure 1:**
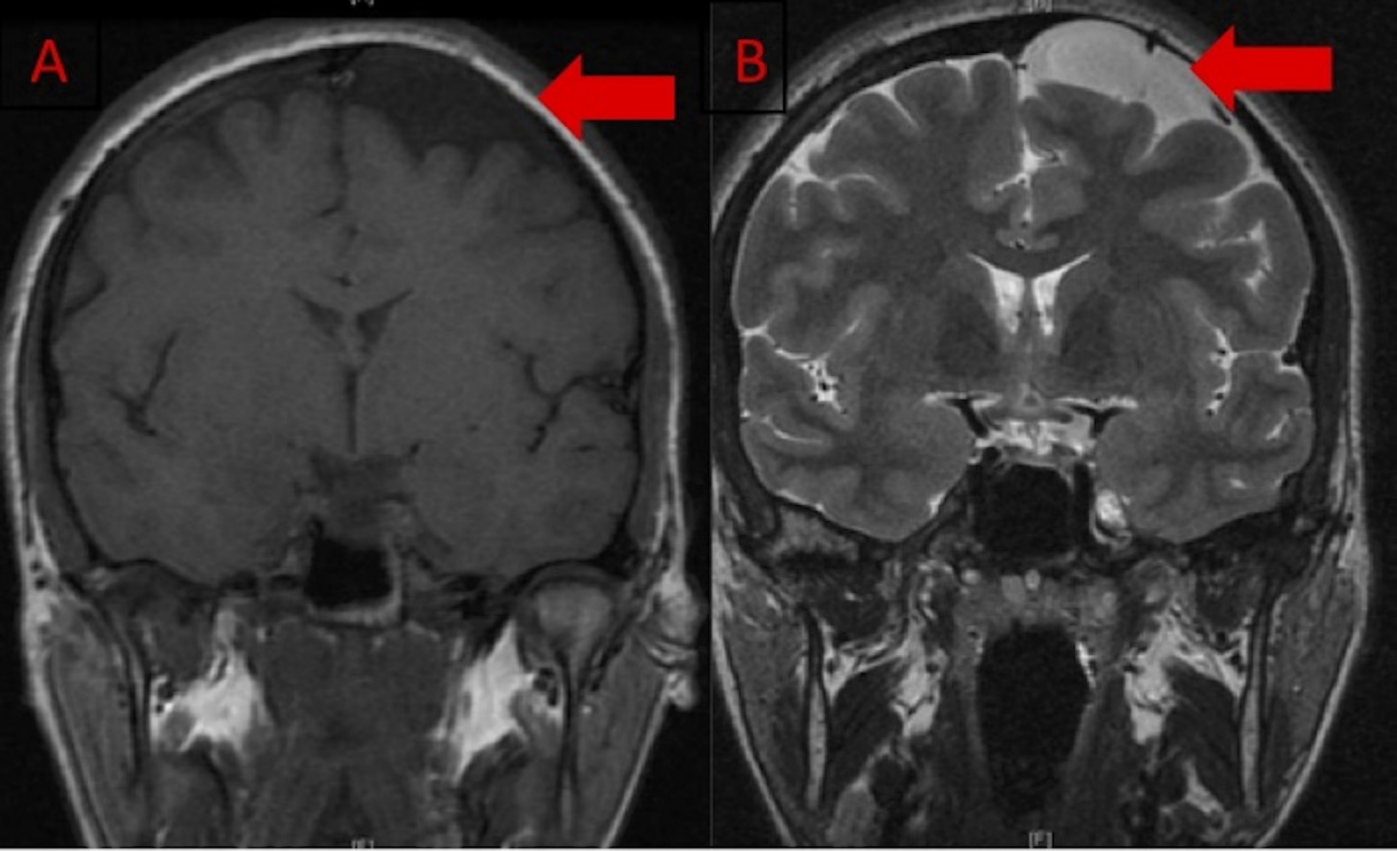
Magnetic resonance imaging of the brain without contrast MRI obtained two years prior to admission. A: Coronal T1-weighted image demonstrating a left frontal hypointense extra-axial lesion. B: Coronal T2-weighted image demonstrating a left frontal hyperintense extra-axial lesion. Both images are suggestive of an arachnoid cyst. MRI: Magnetic resonance imaging

**Figure 2:**
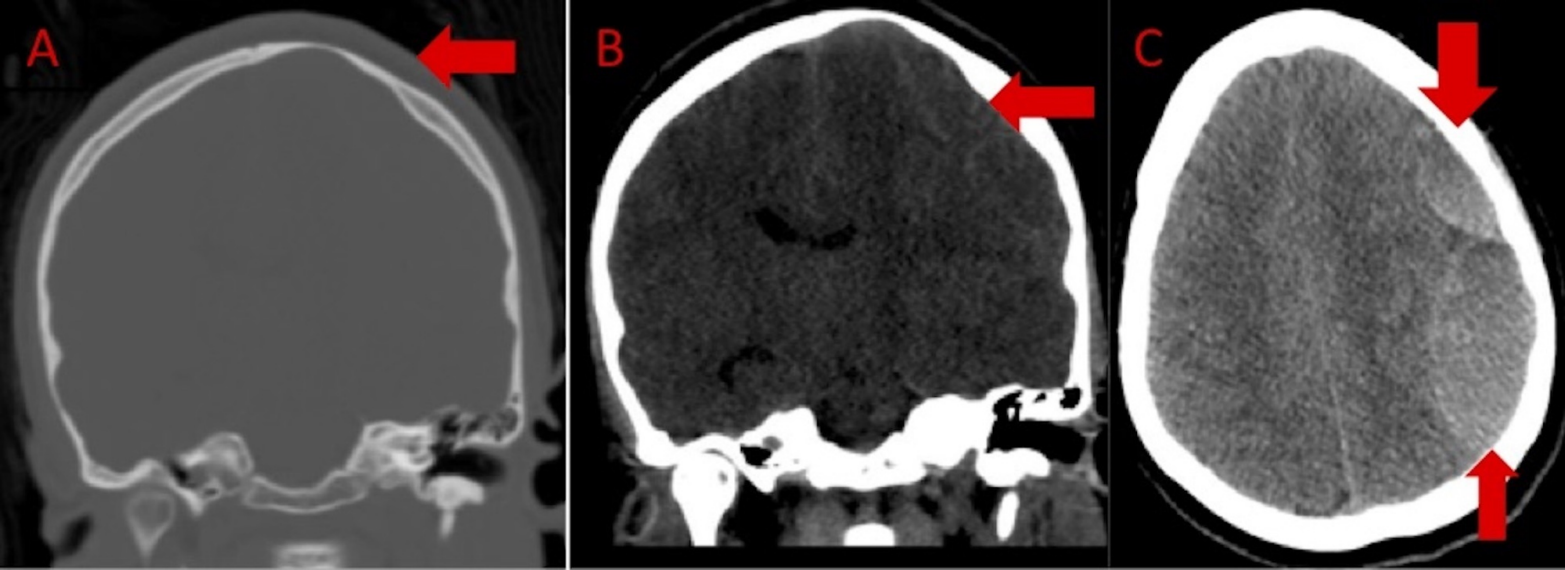
Admission computed tomography of the head A: Coronal view bone window sequence demonstrating scalloping of the calvarium (arrow) in the region of the known left frontal arachnoid cyst. B: Coronal view demonstrating an isodense left frontal extra-axial fluid collection {arrow) with mass effect upon the left cerebral hemisphere with resultant left-to-right midline shift. C: Axial view demonstrating a loculated hyperdense (up-arrow) and isodense extra-axial fluid collection in the region of prior AC, suspicious for intra-cystic hemorrhage (down-arrow) and subdural hematoma.

**Figure 3:**
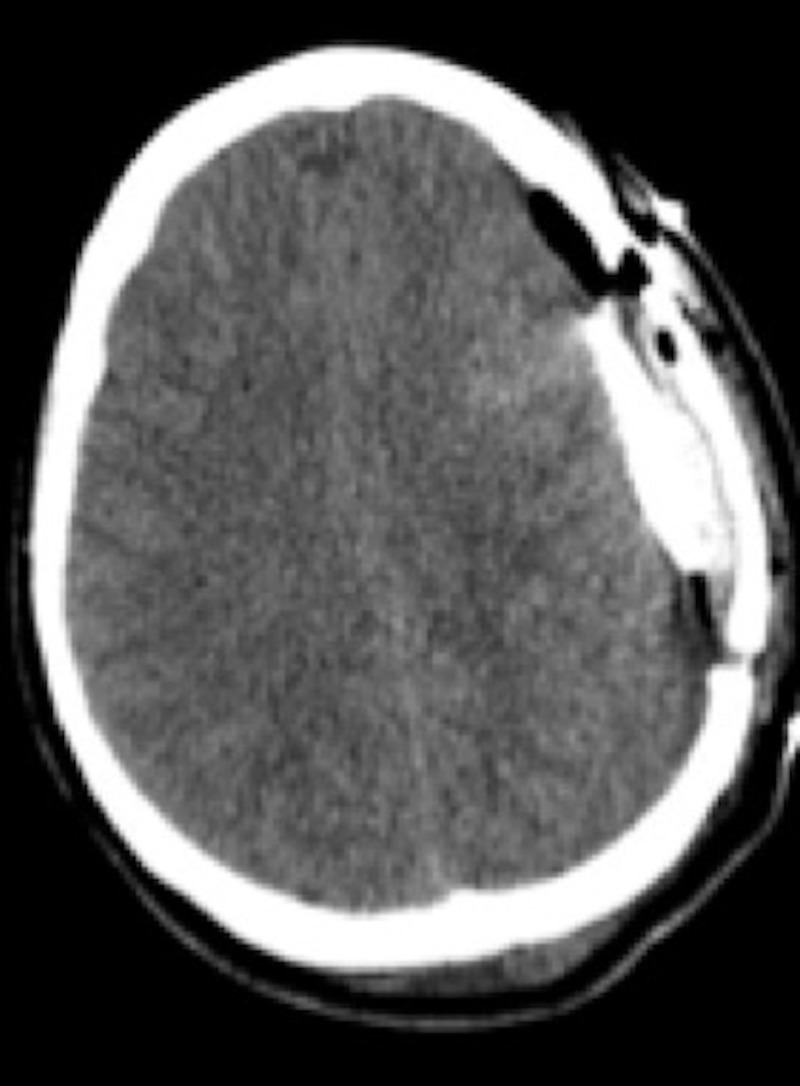
Postoperative noncontrast head computed tomography Axial view demonstrating the evacuation of a subdural hematoma and an arachnoid cyst with intra-cystic hemorrhage. A subdural drain (arrow) was placed intraoperatively to drain the residual subdural hematoma.

**Figure 4:**
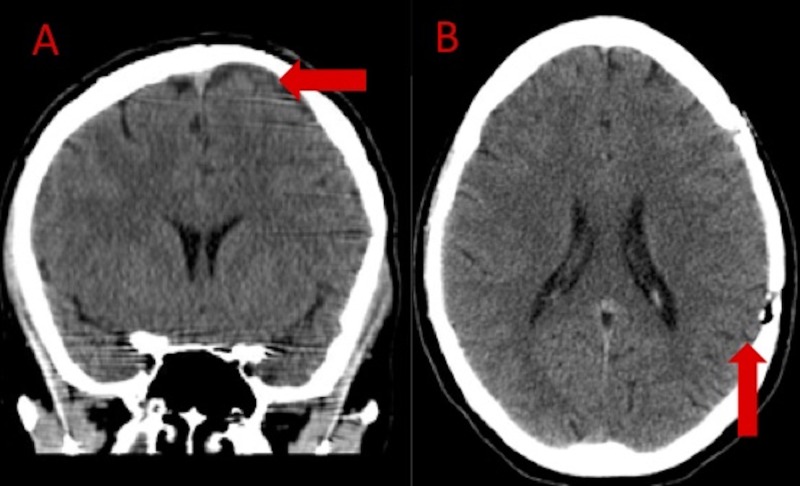
Noncontrast head computed tomography two months after craniotomy A: Coronal view demonstrating resolution of subdural hematoma as well as no residual arachnoid cyst (arrow). B: Axial head computed tomography demonstrating resolution of left cerebral convexity subdural hematoma (arrow).

A 29-year-old Caucasian female with a past medical history of a migraine without aura, a left convexity arachnoid cyst (Figures 1A-1B), and pituitary microadenoma presented to the emergency department with a progressive bifrontal headache. The headaches first started two weeks prior when the patient originally presented to the emergency department and her headache was treated with migraine medication. Her headache initially improved but secondarily worsened, and she started to complain of blurry vision and right arm and leg weakness. A computed tomography (CT) scan of the head was obtained that showed a 2.4 cm left cerebral convexity acute/subacute subdural hematoma (SDH) in the area corresponding to her arachnoid cyst, and associated with a mass effect upon the left frontal and parietal lobes and 6 mm of rightward subfalcine herniation (Figures 2A-2C). The scan additionally showed a hemorrhage within a loculation that suggests an intra-cystic hemorrhage within the arachnoid cyst. The patient denied any history of recent trauma. Neurosurgery was consulted, and the patient was taken to the operating room for a left-sided craniotomy for SDH evacuation. The cyst appeared to be obliterated by the hematoma as there was no evidence of a double arachnoid layer. The patient tolerated the procedure well, and her postoperative head CT showed a successful evacuation of the hematoma and the arachnoid cyst (Figure 3). Her right arm and right leg weakness resolved after surgery. The patient’s postoperative course was uncomplicated, and she was discharged home on postoperative day seven. The patient presented to the emergency department two months after discharge, complaining of a similar headache. Head CT was performed and did not show any re-accumulation of the subdural hematoma or recurrence of the arachnoid cyst (Figures 4A-4B).

## Discussion

Arachnoid cysts (ACs) are congenital, non-neoplastic, extra-axial, intra-arachnoid lesions that contain a fluid similar in composition to cerebrospinal fluid [1-3]. They constitute approximately 1% of all intracranial space-occupying lesions [1,3-5]. Most ACs are indolent lesions that remain asymptomatic until they are found incidentally after neuroimaging for an unrelated problem [4,6-7]; however, ACs can be the source of headaches, increased head circumference, and developmental delay in pediatric patients; rarely, they can cause weakness, seizures, or psychiatric alterations [1]. Complications associated with ACs concern their continued growth, which can exacerbate headaches, create a focal neurological deficit or, particularly with suprasellar and quadrigeminal ACs, also result in obstructive hydrocephalus or brainstem compression, which can be life-threatening [1,8]. The enlargement of ACs can - as in our patient - not only result in the tearing of the bridging veins with subsequent SDHs, but can also result in intra-cystic hemorrhage with a rapid expansion of the AC and the compression of the adjacent structures.  

ACs are increasingly being recognized as a risk factor for the development of chronic subdural hematomas (CSDH) in children and young adults. A case series and review of the literature reported by Xuanxuan et al. described 14 cases of CSDH associated with ACs that required treatment with either burr hole drainage or conventional craniotomy [9]. Six of these patients had no prior history of trauma, and six patients also had intra-cystic hemorrhage at the time of diagnosis. The mean age at presentation in that case series was 13 years, with 13/14 patients 23 years old or younger. Another study published by Yuksel described another case of spontaneous CSDH formation associated with an AC in a 17-year-old male [3]. A third study reported a case series of 241 patients, each with an arachnoid cyst. CSDH associated with an arachnoid cyst occurred in 11 patients, each of which were in the temporal region, leading to the conclusion that temporal ACs are associated with an increased risk of hemorrhage [6]. This study lent support to another study published in 1997 by Parsch et al., who identified that 16/643 cases of CSDH or hygroma were due to a ruptured middle cranial fossa arachnoid cyst [10]. Additional risk factors for AC-associated hemorrhages were studied by Cress et al. in 2013, who published a case-control study that dichotomized the arachnoid cyst size with a cut off of 5 cm in maximal diameter. They found a significantly higher risk of AC-associated hemorrhage in ACs larger than 5 cm in maximal diameter. A history of head trauma was also identified as a risk factor for AC-associated hemorrhage in their study [11].  

Our patient presented with a worsening headache and right-sided weakness secondary to an AC that was complicated by an acute/subacute SDH with an intra-cystic hemorrhage component. Additionally, our patient’s arachnoid cyst was not in the middle cranial fossa, a location that seems to be more prone to AC-associated hemorrhage. The acute, atraumatic SDH our patient developed appears to represent an even more rare sequalae for ACs. Spontaneous intra-cystic hemorrhage and ASDH has been reported by Adin et al. in two patients at their institution [4]. One patient complained of severe headaches and a burning sensation in the left side of his head and face, and the other patient had a one-week history of progressive nausea, vomiting, and headaches. The first case elected for conservative treatment, and the second case underwent a craniotomy. Both patients did well and were discharged without any permanent disability or neurological deficits. Our patient also did well after the surgery, and she was discharged home without any permanent disability or neurological deficit. 

## Conclusions

Arachnoid cysts are typically incidental lesions that are found while the patient is obtaining neuroimaging for unrelated problems. They are benign, extra-axial lesions that usually do not result in neurologic deficits. We presented a unique case of a spontaneous acute SDH and intra-cystic hemorrhage associated with an AC in a 29-year-old female. AC-associated hemorrhage is more commonly found in the setting of recent trauma. Patients with a known AC need to be educated on the potential for both spontaneous or trauma-induced hemorrhage associated with their AC. Current imaging surveillance of these lesions is not standardized. Our institution typically repeats an MRI of the brain between six months to one year to document any growth of the AC. If the AC demonstrates stability, we do not employ any repeat imaging unless clinically indicated. Whether longer imaging surveillance would be beneficial to detect AC-associated hemorrhage and lead to earlier definitive treatment, is a question worth asking and would require studies to evaluate the cost-effectiveness and outcomes of longer imaging surveillance.
